# Secondary Ewing’s sarcoma of the temporo-occipital region developed 12-years after medulloblastoma treatment

**DOI:** 10.1016/j.bjorl.2022.01.003

**Published:** 2022-01-29

**Authors:** Zoran Dudvarski, Nenad Arsovic, Jelena Sopta, Emilija Gacic-Manojlovic, Nemanja Radivojevic, Sasa Jakovljevic

**Affiliations:** a University of Belgrade, Faculty of Medicine, Belgrade, Serbia; b University Clinical Center of Serbia, Clinic of Otorhinolaryngology and Maxillofacial Surgery, Belgrade, Serbia; c Institute of Pathology, Belgrade, Serbia

## Introduction

Modern strategies of multimodal therapy for malignant diseases have significantly increased patient survival rates.^1^ Developing secondary malignant neoplasm is the second leading cause of death in children with an estimated cumulative incidence of 3.3%‒12% over a 20-year follow-up period. Ewing’s Sarcoma (ES) rarely develops as a secondary malignancy, especially in the temporo-occipital region. In most cases, it develops after treatment of primary sarcoma or hematological malignancies.^1^, ^2^

The aim of this study was to present a case of a 23-year-old female patient with secondary ES of the temporo-occipital region that occurred 12-years after medulloblastoma treatment.

## Case report

A 23-year-old female patient was admitted to our clinic due to bleeding from the left ear, pain and swelling of the retroauricular region. Clinical examination revealed the presence of granulations and blood in the left external auditory canal. From the personal history, we learned that the patient had undergone surgery for medulloblastoma 12-years ago, after which there were remaining signs of peripheral facial nerve palsy (House-Brackmann score III) and cerebellar syndrome. The patient received TD 20 Gy in the head region and TD 35 Gy in the spinal region. In chemotherapy, VI cycles were applied by the PECKER protocol ‒ Lomustine (CCNU), Vincristine (VCR) and Cisplatin (CDDP).

**Figure 1:**
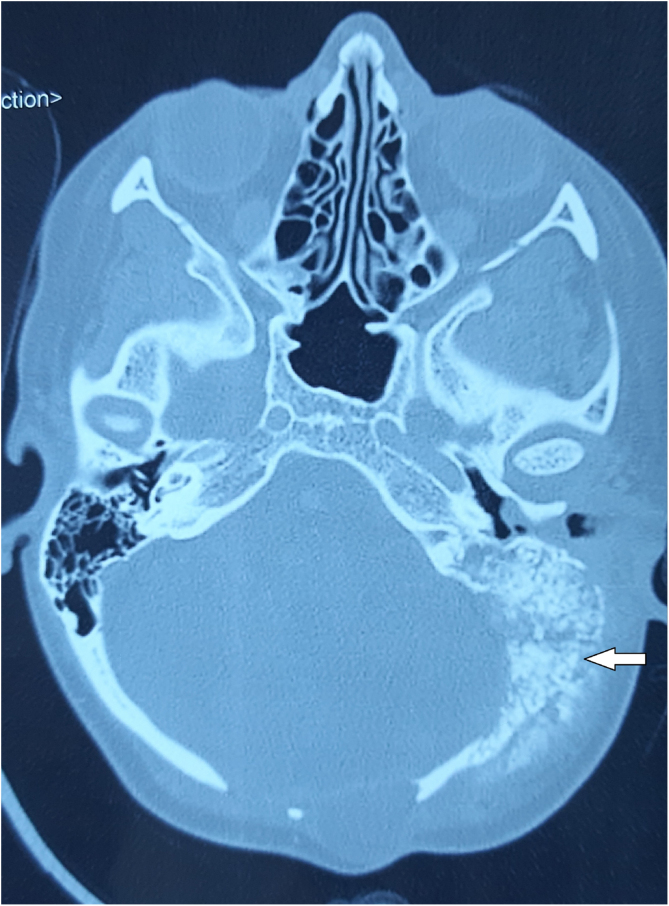
CT of the temporal bone (axial section). Sclerotically altered and destroyed left occipital and temporal bone with the formation of new bone (white arrow).

Computed Tomography (CT) of the temporal bone revealed sclerotically altered and destroyed left occipital and temporal bone with signs of inflammation in mastoid cells (Fig. 1).

Magnetic Resonance Imaging (MRI) of the endocranium indicated extensive post-treatment sequelae in the cerebellum region, with no clear signs of the tumor recurrence. Pathological content was present in the mastoid cells and tympanic cavity with the structurally altered left temporal and occipital bone.

**Figure 2:**
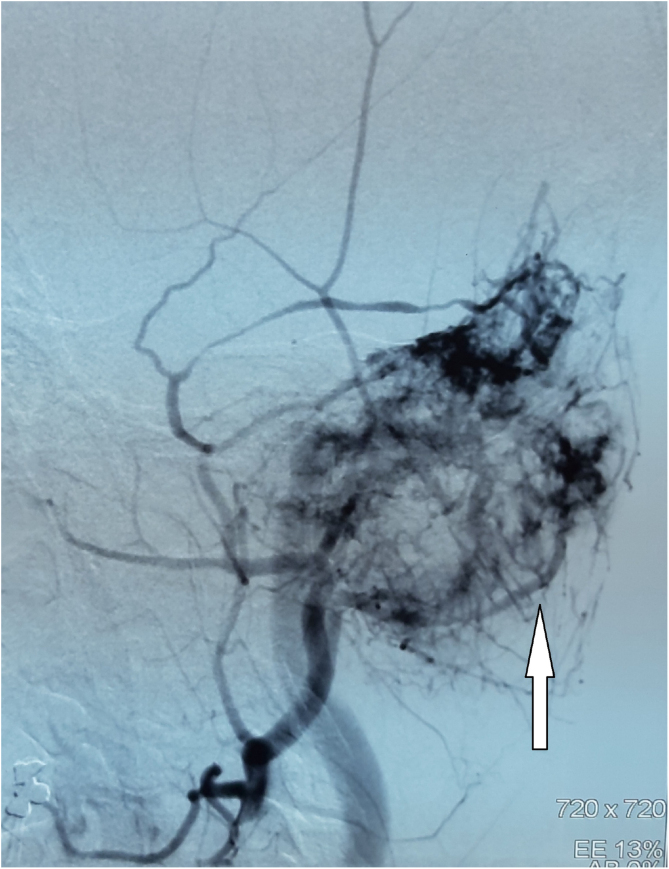
DSA indicates a hypervascular tumor of the left temporo-occipital region (white arrow).

Digital Subtraction Angiography (DSA) showed a hypervascular tumor in the projection of the left temporal bone with a pronounced fistulous component. The tumor was irrigated from the left a.carotis externae, the dural branches of the left a.carotis internae, as well as the dural branches of the left vertebral artery (Fig. 2).

Chest CT scan indicated numerous, predominantly calcified, micro- and macronodular changes in the lungs, and osteoblastic deposit on the Th1 vertebral body.

Ultrasound examination of the abdomen was normal.

Tumor mass was biopsied by retroauricular approach under general anesthesia. Intraoperatively, the mastoid cortex was destroyed, and when taking a biopsy of soft tissue tumor from the mastoid cavity, profuse venous bleeding occurred, which was difficult to control.

**Figure 3:**
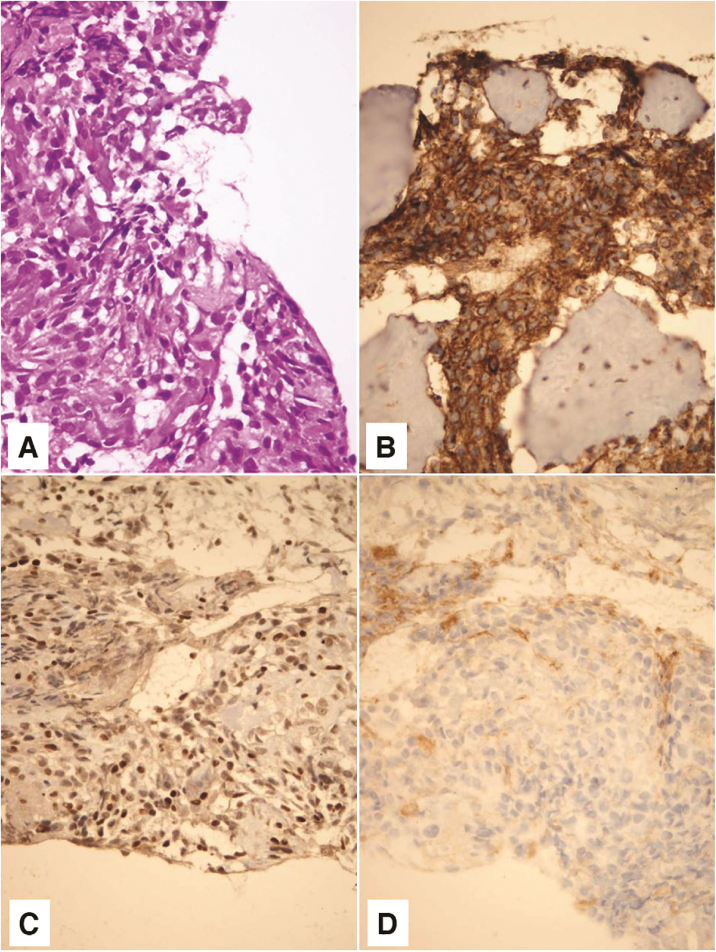
On hematoxylin - eosin staining, tumor was composed of small, poorely differentiated cells with hyperchromatic nuclei and scant cytoplasm (A). Tumor cells showed membrane positivity for CD 99 (B) and nuclear positivity for FLI1 (C). Negative staining for CD 31 tumor cells ruled out the possibility of a vascular lesion (D). Magnification ×400.

Pathohistological and immunohistochemical analysis showed that the tumor corresponded to a high-grade secondary mesenchymal tumor from the Ewing’s sarcoma group (Fig. 3). Tumor cells were positive for CD 56 (diffusely, intensely), Synaptophysin (focally, moderate intensity), CD 99 (diffusely, intensely), and FLI (diffusely, variable intensity). Tumor cells were negative for Neuronal nuclear protein (NeuN), Glial Fibrilary Acidic Protein (GFAP), Cluster of differentiation 31 (CD31), and Avian v-ets erythroblastosis virus E26 oncogene homolog (ERG). NeuN and GFAP are neuronal (former) and glial (later) markers which can be focally expressed in medulloblastoma. Subsequently, they were used to exclude the diagnosis of medulloblastoma. ERG is a marker which can be expressed in a subset of Ewing sarcoma, characterized by the second most common molecular abnormality, EWSR1-ERG fusion. Thus, ERG was used in order to potentially recognize a rare form of Ewing sarcoma. Fluorescence In situ Hybridisation (FISH) analysis was not done for technical reasons, as profuse bleeding during the biopsy prevented taking a larger amount of material for further analysis.

The patient was presented to the Oncology Council which decided to start treatment with VIDE (Vincristine, Ifosfamide, Doxorubicin, Etoposide) protocol. On the first day, the therapy was discontinued due to breathing problems, and a new chest CT scan showed a massive progression of metastatic lung disease. Unfortunately, the patient passed away soon after.

## Discussion

The development of ES in patients treated for other malignancies has been described in the literature, usually in the form of case reports or short series.^1^, ^3^ Wolpert et al. reported in 2016 that only 38 cases of secondary ES had been described after hematological malignancy treatment.^1^ Secondary ES most commonly occurred in the chest wall and long bones, and only one case developed in the head and neck region and that in the mandible.^1^

In the largest series of secondary ES, the time elapsed from the primary malignancy diagnosis to the secondary ES diagnosis was on average 64-months.^2^ Similar data were presented by other authors, with a latency of 1‒17-years.^1^, ^3^ In our case, the latent period between the primary medulloblastoma treatment and secondary ES diagnosis was 12-years, which is in line with the data from the literature.

Based on the latest findings, ES is assumed to be of neuroectodermal origin. The very degree of neuronal differentiation itself was used to histopathologically subclassify tumors from the ES family, using standard and electron microscopy or immunohistochemistry with neuronal markers, including neuron-specific enolase, Leu-7, or synaptophysin. Overexpression of CD 99, a transmembrane protein encoded by the MIC-2 gene, is one of the characteristics of this tumor.^4^ In our patient, CD 56, Synaptophysin, CD 99, and FLI were positive, while tumor cells were negative for NeuN, GFAP, CD 31, and ERG.

ES are characterized at the molecular level by the existence of a translocation between the EWSR1 gene and a member of the ETS gene family resulting in the translocation of t(11;22)(q24;q12)(EWS-FLI1).^2^, ^5^ Molecular genetic analysis (FISH analysis) for this translocation is pathognomonic of Ewing’s sarcoma. As noted above, the FISH analysis was not performed on our patient for technical reasons as there was profuse bleeding from the tumor during the biopsy and the amount of material to be analyzed was insufficient.

Risk factors leading to secondary malignant neoplasms include age, hereditary predisposition, and side effects of primary malignancy therapy.^5^ According to Ishida et al. patients who underwent cranium RT were at 6-times higher risk for secondary malignancies development. Interestingly, only a small number of secondary ES described in the literature developed at the site within the X-Ray field for primary malignancy.^1^, ^2^, ^3^ Our patient received a total TD 55 Gy in the head and spine region, including the temporo-occipital region, where later secondary ES developed.

Alkylating agents increase the relative risk of secondary malignant neoplasms by 1.4‒2.2 times.^3^ Our patient was previously treated for medulloblastoma with VI cycles of PECKER protocol chemotherapy.

Applebaum et al. state that in patients with secondary ES the 5-year overall survival is 34.3% compared with 52.2% for patients with primary sarcoma.^2^ About 20%‒25% of patients with primary or secondary ES have metastases present at the time of diagnosis and have poor prognosis.^4^ At the time of diagnosis, our patient had already developed numerous metastases in the lungs and bones, and after starting the VIDE chemotherapy protocol, there was disease worsening and lethal outcome.

## Conclusion

Although modern malignant tumor treatment has significantly improved survival rates, there is a need for careful identification of patients at risk of developing secondary malignancy. Secondary ES of the temporal bone is extremely rare but must be considered in the differential diagnosis of hypervascular lesions in patients previously treated for malignancy.

## Conflicts of interest

The authors declare no conflicts of interest.
